# The LIVES Daily Hassles Scale and Its Relation to Life Satisfaction

**DOI:** 10.1177/10731911211047894

**Published:** 2021-10-18

**Authors:** Shagini Udayar, Ieva Urbanaviciute, Davide Morselli, Grégoire Bollmann, Jérome Rossier, Dario Spini

**Affiliations:** 1 Institute of Psychology,University of Lausanne, Lausanne, Vaud, Switzerland; 2 Institute of Social Sciences, University of Lausanne, Switzerland; 3 Swiss National Centre of Competence in Research LIVES, University of Lausanne, Switzerland; 4 Department of Psychology, University of Zurich, Zurich, Switzerland

**Keywords:** daily hassles, LIVES Daily Hassles Scale, life satisfaction, scale development

## Abstract

Although daily hassles have been of interest since the 1980s, only a few tools have been developed to assess them. Most of them are checklists or open-ended questions that are demanding for participants in panel surveys. Therefore, to facilitate daily hassles integration into large surveys, the aim of this study was to present a new tool assessing daily hassles, the LIVES–Daily Hassles Scale (LIVES-DHS), and to examine its relation to life satisfaction, in a sample of 1,170 French- and German-speaking adults living in Switzerland. In a first random subsample, we conducted a principal axis factor analysis, and the results suggested a five-factor solution. Furthermore, we conducted a confirmatory factor analysis on a second random subsample, and it supported the hierarchical factor structure of the scale. The LIVES-DHS consists of 18 items represented by five factors that describe five sources of daily hassles: financial, physical, relational, environmental, and professional. The bivariate correlations showed that the LIVES-DHS could differentiate the concept of daily hassles from associated concepts. Finally, the hierarchical regression showed that daily hassles negatively predicted life satisfaction and added a significant incremental variance beyond that accounted for by age, gender, household income, education level, and personality traits.

Major life events such as marriage, death of a loved one, or unemployment are likely to have an impact on almost all aspects of daily life (e.g., Luhmann et al., 2012; Perrig-Chiello et al., 2016). However, their cumulative effect on health and well-being may be not as large as that of minor yet frequent reoccurring events, such as daily conflicts at home or at work (Lazarus & Folkman, 1984). Daily hassles are defined as routine nuisances of day-to-day living (Kanner et al., 1981). They refer to unexpected small occurrences that disrupt daily life. When repeated with some frequency over time, such disruptions may impair health and well-being (e.g., Day et al., 2005; Graf et al., 2017; Hart 1999).

To investigate people’s recurrent exposure to stressors such as daily hassles, a few tools have been developed and implemented (e.g., DeLongis et al., 1988; Volmer & Fritsche, 2016). They usually take the form of exhaustive checklists or open-ended questions. However, such tools are demanding for participants in large surveys in which administration time is a major issue. The aim of this study is to present a new short tool, the *LIVES–Daily Hassles Scale* (LIVES-DHS), for measuring daily hassles. Contrary to other existing tools that tap into the presence/absence, frequency, or intensity of daily stressors (e.g., DeLongis et al., 1988), our proposed questionnaire measures individual concerns about a number of potential daily hassles in different domains based on Lazarus and Folkman’s (1984) concept of primary appraisal from their transactional model of stress and coping. Hence, this study presents not only a new scale but also a new way of assessing daily hassles.

## Daily Stressors

Researchers considered daily minor stressors for the first time when studying stress and health in the 1980s (DeLongis et al., 1982). A broad spectrum of everyday hassles might occur on a daily basis: conflicts with the partner or family, financial issues, traffic jams, dealing with annoying customers, and so on. Therefore, daily hassles are by definition rather recurrent and might be context specific (e.g., for work context, see Silva & Caetano, 2013; Klusmann et al., 2020). However, stressors occurring in one specific domain (e.g., conflicts with partner in the family context) can also spill onto another domain (e.g., negative mood that creates conflicts with colleagues at work). This phenomenon is known as spillover effect (Pearlin & Bierman, 2013), and it partly explains why daily hassles that occur in a specific context can affect different aspects of one’s life.

A potential hassle becomes a hassle if it is appraised as such and not simply because it occurs (Larsson et al., 2016). This idea has been first developed by Lazarus and Folkman (1984) in their cognitive appraisal theory, which highlights the role of relatively minor stressors that characterize everyday life. This theory conceives stress as an active process in which the joint effects of the characteristics of an event and the characteristics of the person on health and well-being are considered. A daily event turns into a hassle only when the people evaluate it as a threat, against which they do not have sufficient resources.

## Measuring Daily Hassles

Several tools for measuring the exposure to and severity of daily hassles have been used to date, such as structured checklists or open-ended questions, with different modes of data collection (e.g., surveys, dairy studies, and interviews). The advantage of checklists is that they are easier and faster to answer, resulting in a lower load of work for the respondents. However, this method may fail to capture certain daily stressors if they are not included in the list, and it is often difficult to assess how important a hassle is for the person from a subjective perspective. Moreover, checklists that are too long may negatively affect respondents’ attention and increase dropout or biased responses (e.g., Krosnick, 1999).

Almost all existing daily hassles assessments use the checklist methodology, which makes them difficult to implement in large surveys. For example, the 320-item Unpleasant Events Schedule was one of the first checklists developed to assess the frequency and subjective aversiveness of daily events in seven domains: health and welfare, achievement-academic-job, domestic inconveniences, sex-marital-friendship, material-financial, and social exits (Lewinsohn & Talkington, 1979). At the same period, Stone and Neale (1982) developed the Assessment of Daily Experience, a 63-item checklist including daily events distributed across five major domains: work-related, leisure, financial, family and friends, and other. Researchers also developed a 117-item Hassles Scale (53 items in the revised version) evaluating the frequency and intensity of the hassles and covering the domains of work, health, family, friends, environment, practical considerations, and chance occurrences (DeLongis et al., 1988; Kanner et al., 1981). Finally, some years later, researchers developed the 58-item Daily Stress Inventory assessing the intensity of minor stressful events (Brantley et al., 1987) and a much shorter 21-item checklist (Bolger et al., 1989) assessing the occurrence of daily hassles in different domains (i.e., overload at home or work, family and other demands, transportation problems, financial problems, and interpersonal conflicts and tensions). A number of similar tools have been designed in psychology to assess hassles generally or in specific domains such as the work domain (e.g., Basch & Fisher, 2000).

Open-ended questions to assess daily hassles have also been created as an alternative to checklists (for work events, see Volmer & Fritsche, 2016). Although easier to implement in surveys, they have the disadvantage of being more demanding for both the respondents and researchers. With such a method, respondents are required to recall the events that happened during a certain period (e.g., day or month) and describe them in several sentences. It is therefore a task that can be time consuming and cognitively demanding. For the researchers, it implies a long coding process and may be challenging and subject to interpretation, especially because answers may differ in level of detail, although some techniques have been recently developed to analyze automatically these open-ended questions (Zhang et al., 2019).

To resolve the aforementioned issues of checklists and open-ended questions, some recent studies have developed an alternative approach that combines both of them (e.g., for daily work events, see Kuba & Scheibe, 2017; Schmidt et al., 2017). For example, Schmidt et al. (2017) assessed daily hassles by asking individuals first to report up to 10 daily events in an open-ended format and then rate the valence of these experiences using a Likert-type scale (from negative to positive). Subsequently, trained raters sorted these events into categories. Klusmann et al. (2020) used a similar method; interestingly, as an operationalization of primary appraisal, their participants rated the subjective relevance in addition to the valence of the events. As such, open-ended questions may be a viable alternative in smaller scale studies. However, the lack of standardization and systematic evaluation of potential stressors makes them unsuitable for large panel studies. Moreover, the required categorization of open-ended questions makes such solutions very costly and time-consuming with the current method of coding.

For these reasons, at the Swiss National Centre of Competence in Research LIVES, we developed a LIVES-DHS as a self-reported questionnaire for measuring daily hassles that can be implemented in large surveys. Short enough but still assessing different stressors, this new questionnaire was inspired by existing literature on the topic, tapping five sources of daily stress: financial, physical, relational, environmental, and professional. Different from existing daily hassles inventories that count daily events from an exhaustive list, the LIVES-DHS aims to assess different sources of daily hassles not by asking about their frequency but inquiring about how much they are a preoccupation for the respondents. Hence, the aim of this new scale is to measure subjective experiences of daily hassles. In other words, the LIVES-DHS aims to measure individual concerns about a number of potential daily hassles, drawing on the assumption that hassles become hassles when they are perceived as such and not merely by their presence or absence in everyday life.

### Personal Characteristics and Daily Hassles

Some personal characteristics may help identify which individuals are subject to stressful situations and which are not. For example, an individual’s personality may play an important role in the stress process (Lazarus, 2006). It has been shown that the personality dimension of neuroticism may increase the likelihood of experiencing daily hassles and reacting negatively to them by increasing the recall of negatively toned information (e.g., Bolger & Schilling, 1991; Suls & Martin, 2005). Similarly, gender seems to relate to individual differences in the experience of daily hassles. Indeed, women report more frequent daily stressors and rate stressors as more severe than do men (e.g., Almeida et al., 2002; McIntyre et al., 2008). Finally, age also seems to relate to individual differences in the exposure and appraisal of daily hassles although the results are mixed (e.g., Aldwin et al., 2014; Neubauer et al., 2019; Stawski et al., 2008)

### Blurry Boundaries Between Daily Hassles, Stress, and Poor Mental Health

One of the challenges when studying daily hassles is to delineate the boundary of daily hassles, stress, and poor health and well-being (Wright et al., 2020). Care should be taken in distinguishing between stressors (i.e., daily hassles), appraisals of them (e.g., perceived stress, anxiety, and distress), and strains (e.g., psychological or health outcomes; Bliese et al., 2017). Otherwise the relations between these key elements could be artificially inflated because of unclear conceptual boundaries or measurement confounding. Specifically, it is important for a daily hassles scale to distinguish the events from the appraisal of, and response to them. For example, it has been shown that global perceived stress (i.e., individuals’ feelings of their life situations as stressful) is only low to moderately correlated to daily hassles (e.g., Cohen et al. 1983; van Eck et al., 1998), which suggests that the two scales assess different parts of the stress process. Moreover, it is also important for such scales to differ from key-related outcomes. For example, regarding the LIVES-DHS, each source of daily hassles (i.e., financial, physical, relational, environmental, and professional) could be closely related, yet distinct, from domain specific indicators of strain, such as income adequacy, self-rated health, and job insecurity, or resources, such as social support. Income inadequacy, poor self-rated health, or job insecurity may be seen as outcomes of financial, physical/health, and professional/work-related daily hassles (e.g., Vásquez et al., 2019; Wright et al., 2020). Social support, which represents the positive aspect of interpersonal relations, is substantively distinct from social conflict, which is one of the main sources of relational hassles. When individuals report on social support and social conflict, they most likely think of different people (i.e., friends vs. foes). Hence, facing relational hassles does not necessarily mean having less support from others.

### Daily Hassles, Health, and Subjective Well-Being

For over 40 years, scholars have examined the relation between daily hassles and mental and physical health mainly through cross-sectional and correlational studies and the use of inventories. Hence, daily hassles is a well-known risk factor for health and well-being, even beyond the effect of major life events (e.g., DeLongis et al., 1982; Zarski, 1984). More specifically, research has highlighted their association with psychological symptoms (Kanner et al., 1981; Lu, 1991), psychological distress (Serido et al., 2004), mental disorders (Asselmann et al., 2017), such as depression (Bouteyre et al., 2007; Lewinsohn & Talkington, 1979; McIntosh et al., 2010) and anxiety (Réveillère et al., 2001), and health problems such as headaches (DeLongis et al., 1988; Fernandez & Sheffield, 1996; Graf et al., 2017). Other studies have revealed that daily hassles are related to exhaustion and burnout (Fritz & Sonnentag, 2005; Klusmann et al., 2020; Schmidt et al., 2017). Overall, studies have shown that both the frequency and intensity of daily hassles are related to physical and psychological health (e.g., Fernandez & Sheffield, 1996; Ivancevich, 1986) and quality of life (e.g., Strenna et al., 2014).

However, to date research has primarily focused on health-related outcomes and only a few have looked into subjective well-being (term used for scientific exploration of happiness; Diener, 1984). For example, Hart (1999) found that daily hassles predicted overall nonwork satisfaction within a police officer sample. Day et al. (2005) showed that daily hassles were a predictor of life satisfaction. Lavee and Ben-Ari (2008) found that daily hassles were related to life satisfaction in both individualist and collectivist cultural orientation populations. Finally, Wright et al. (2010) revealed that daily hassles had a negative association with life satisfaction within an adolescent sample. Overall, although these studies established the association between the two constructs, none of them examined the effect of daily hassles on life satisfaction controlling for personality traits that are a well-established covariate of life satisfaction (Erdogan et al., 2012: Steel et al., 2008).

Because previous researchers have pointed out a relation between daily hassles, health, and well-being, the development of a new scale adapted to research designs like panel surveys is essential in increasing our understanding of the extent to which daily hassles from different domains impact one’s health and well-being. To this goal, a short and reliable measure assessing daily hassles is necessary but lacking in the survey panorama.

### The Present Study

The aim of this study was to present and validate the final version of the LIVES-DHS. Hence, we first examined the psychometric properties of the scale (i.e., reliability and structure applying an exploratory factor analysis [EFA]) by using half of the sample. Then, we conducted a confirmatory factor analysis (CFA) to cross-validate the structure using the other half of the sample. Given the conceptual considerations outlined above, the scale we developed and previous studies (Bolger et al., 1989; DeLongis et al., 1988; Kanner et al., 1981), we hypothesized the following:

**Hypothesis 1a:** A five-factor model of daily hassles would fit better than a one-factor model.
**Hypothesis 1b:** Moreover, based on the stress spillover process, the five sources of daily hassles would be highly correlated so that they form a general higher order factor (internal validity hypothesis).

In a subsequent step, we examined the concurrent validity of the LIVES-DHS. We examined concurrent validity by computing bivariate correlations (a) between the different sources of daily hassles and theoretically relevant constructs; namely, income adequacy, self-rated heath, social support, and job insecurity; (b) between the overall daily hassles score and overall/global indicators of stress and health such as perceived stress in life and general mental health, and (c) between the overall daily hassles score and personality dimension of neuroticism.

**Hypothesis 2a:** The different sources of daily hassles would yield significant but moderate correlations with income adequacy, self-rated heath, social support, and job insecurity, indicating that LIVES-DHS could differentiate the sources of daily hassles from associated concepts.
**Hypothesis 2b:** Daily hassles (overall score) would yield significant but moderate correlations with perceived stress in life and general mental health, indicating that LIVES-DHS could differentiate the concept of daily hassles from general outcomes related to those events.
**Hypothesis 2c:** Daily hassles (overall score) would yield significant but moderate correlations with neuroticism, indicating that LIVES-DHS could differentiate the concept of daily hassles from a predisposition to experience daily hassles.

Finally, we examined the relation between daily hassles and life satisfaction using a series of hierarchical regressions that assessed whether daily hassles adequately predicted life satisfaction beyond age, gender, household income, education level, and personality traits.

**Hypothesis 3:** Daily hassles, both overall measure and each source of daily hassles, would predict a lower level of life satisfaction controlling for age, gender, household income, education level, and personality traits.

## Method

All data and syntax necessary to reproduce the reported analyses are publicly accessible on the Open Science Framework: https://osf.io/6zvud/. A priori calculations using Soper’s (2019) SEM sample size calculator suggested a minimum sample size of 376 was required for analysis to yield adequate power. This was based on a small-medium anticipated effect size (0.2), 5 latent variables, 22 observed variables, and a desired power level of 0.8 at a probability level of 0.05.

### Participants

We conducted the analyses on an adult sample of the French- and German-speaking Swiss population (*N* = 1,170), aged between 31 and 61 years (*M*_age_ = 47.92, *SD* = 8.31), living in Switzerland. Women represented 51.5% of the sample, and German speakers represented 64.6%. Participants were mostly employed (89%), whereas the remaining sample was either unemployed (4.4%) or professionally inactive (6.6%). The majority of participants lived in a couple (71.6%), and 28.4% were single.

### Procedure

We implemented the LIVES-DHS in Wave 6 (2017) of a data collection from a 7-year longitudinal study on professional paths conducted at the Swiss National Centre of Competence in Research—Overcoming Vulnerabilities: Life Course Perspective (NCCR LIVES). The aim of this project was to study the direct and indirect impacts of individual characteristics and resources, and sociocultural and economic background on adults’ professional transitions.

This panel study was conducted from 2012 (W1) to 2018 (W7) with an annual assessment. Participants were recruited at W1 on the base of a representative sample (26–56 years) of the German-speaking and the French-speaking regions randomly drawn from the National register of the inhabitants and realized by the Swiss Federal Statistics Office. To increase the number of unemployed participants, a second random sample was realized by the State Secretariat for Economic Affairs and was drawn from the national register of unemployed people. In total, 2,469 individuals fully completed the questionnaire at W1.

Each year participants completed a research protocol consisting of two steps. The first part assessed the participants’ professional situation and biographical information. Based on the answers to this part, participants were classified into three categories representing their professional situation: active (i.e., employed), unemployed, or nonactive on the labor market. The second part, which depended on the participants’ professional situation, assessed aspects of the work environment (e.g., job strain, job insecurity, and work stress), personal characteristics and resources (e.g., character strengths, personality traits, and career adaptabilities) and general outcomes (e.g., health, life satisfaction, and quality of life).

Participation in this panel study was voluntary; each participant completing the research protocol could choose one of four gifts for an amount of 20 CHF, yearly. The full description of the study and the data are stored in the FORSbase data repository and are available on request (https://forsbase.unil.ch/project/study-public-overview/16093/0/).

### LIVES Daily Hassles Scale Development

A group of experts (sociologists and psychologists) in life course studies identified different life domains where daily hassles could be perceived based on the aforementioned literature. They then created a pool of items in French and German inspired by existing daily hassles inventories mentioned in the theoretical part. The items were created in French first. The scale was then translated by one native German speaker, and the translation checked and corrected by a second German native speaker. Differences were discussed and resolved between the two translators. Respondents had to indicate the extent to which a series of possible daily hassles concerned them on a 5-point Likert-type scale (1 = *not at all*, 2 = *a little*, 3 = *it does not concern me*, 4 = *somewhat*, 5 = *very much*). The initial questionnaire comprised 23 items and tapped five different life domains: finance, health, relations, work, and environment. This version of the scale was subsequently included in two independent surveys (both used representative Swiss samples of adults, but one focused mainly on people aged older than 65 years) conducted within the NCCR LIVES. The results of EFA conducted with these samples indicated that one item about transportation (i.e., having to deal with problems related to daily travel, such as travel time or traffic) from the environmental stress domain should be dropped because it failed to meet the minimum criteria of having a primary factor loading of .4 or above. The results also showed some issues with scale responses, especially for the German version. More precisely, the response category “it does not concern me,” which had to be recorded as missing for the analyses, biased the results of group comparisons. These unsatisfactory results compelled the group of experts to readjust the scale by excluding one item and use a 4-point Likert-type response scale instead (1 = *not at all*, 2 *= little*, 3 = *somewhat*, 4 = *very much*). This adjusted 22-item scale was tested again in a third sample of employed Swiss adults aged between 20 and 65 years. Because the scale showed better psychometric properties this time, it was implemented in our 7-year longitudinal study on professional paths.

However, as the domain of relational sources of daily hassles was composed of many items (7 in total), we decided to further drop items that were relevant only to people in a relationship (i.e., having to deal with serious conflicts with partner or spouse; vs. being alone, without a partner or spouse). Hence, the final set of items used for the validation study described below comprised 20 items.

### Measures

#### Daily Hassles

We assessed daily hassles using the LIVES-DHS at W6. After validation and final item reduction described further below, the final version comprises 18 items using a 4-point Likert-type response scale (1 = *not at all*, 2 = *a little*, 3 = *somewhat*, 4 = *very much*). It covers five sources of daily hassles: financial, physical, relational, environmental, and professional (see Appendix 1, available in the online supplement). Respondents had to rate to what extent they worried about a number of different sources of preoccupations on a given day. Items 17 and 18 were only given to employed participants, as they are not relevant to unemployed or professionally inactive people. The Cronbach’s alpha coefficient of this final version was .95 (for the entire scale), and it was .87, .87, .81, .88, and .92 for the five sources of daily hassles, respectively.

#### Income Adequacy

We assessed perceived income adequacy at W6 with a one-item scale adapted from a similar item in the Eurostat survey (i.e., in an average month, how easy or difficult is it for you to make ends meet and pay all your bills?). More specifically, participants had to report how difficult it is to get by with their current income using a 4-point Likert-type scale (1 = *very difficult*, 4 = *very easy*).

#### Self-Rated Health

We measured self-rated health at W6 using one item developed and used by the World Health Organization (Skevington et al., 2004). We asked the participants to rate their health in general on a 5-point Likert-type scale (1 = *very bad*, 5 = *very good*).

#### Perceived Social Support

We used the eight-item Duke-UNC Functional Social Support scale (Broadhead et al., 1988) to measure individuals’ perception of social support available from others (assessed at W6). The item response options were on a 5-point scale ranging from 1 (*much less than I would like*) to 5 (*as much as I would like*). Cronbach’s alpha coefficient for the total score was .93. The structural validity of this scale was analyzed in a previous study using data from the same panel study (Udayar et al., 2020a).

#### Job Insecurity

We measured job insecurity at W6 using three items specifically designed for the study. These items assess key aspects of job insecurity as suggested by well-known job insecurity models and measures (e.g., Borg & Elizur, 1992; Probst, 2003). The first item measures the satisfaction with job security (1 = *very satisfied*, 4 *= very dissatisfied*), the second item pertains to the fear of job loss in the upcoming 12 months (1 = *do not fear at all*, 4 *= highly fear*), and the third item taps into a cognitive evaluation of job security (i.e., “My job security is good”; 1 *= totally agree*, 4 *= totally disagree*). A composite indicator of job insecurity was derived based on the mean score of these items, where a higher score indicates higher job insecurity. Cronbach’s alpha was .82. In a previous study using data from the same panel study, measurement equivalence across different time points and factor loading invariance across the French- and German-speaking subsamples were observed (Urbanaviciute et al., 2019a).

#### Personality Traits

We used the 60-item NEO Five-Factor Inventory–Revised (McCrae & Costa, 2004) to measure the five main personality dimensions proposed by the five factor model: neuroticism (N), extraversion (E), openness to experience (O), agreeableness (A), and conscientiousness (C). Contrary to all the other measures, we assessed personality traits in Wave 4, using 12 items for each personality dimension. The response format was a 5-point Likert-type scale of 1 (s*trongly disagree*) to 5 (*strongly agree*). The Cronbach’s alpha coefficients for the five dimensions were .86, .74, .77, .72, and .79, respectively. The structural validity of this scale was analyzed in a previous study using data from the same panel study (Udayar et al., 2020a).

#### General Mental Health

We used the 12-item version of the General Health Questionnaire (Goldberg & Williams, 1988) to assess mental health complaints at W6. Respondents had to evaluate their occurrence on a 4-point response scale (i.e., better than usual, same than usual, less than usual, and much less than usual). As suggested by several authors, we used a modified dichotomous coding system (0–1–1–1), called the Goodchild and Duncan-Jones’s (1985) method. The Cronbach’s alpha was .91.

#### Perceived Stress in Life

We used the five-item Perceived Stress Scale (Cohen et al., 1983) at W6 to measure the degree to which situations in one’s life have been appraised as stressful over the period of last month. Respondents had to indicate how unpredictable, uncontrollable, and overloaded they found their lives to be (e.g., “How often have you felt that you were unable to control the important things in your life?”) using a 5-point Likert-type scale of 1 (*never*) to 5 (*very often*). The Cronbach’s alpha was .71. The structural validity of this scale was analyzed in a previous study using data from the same panel study (Urbanaviciute et al., 2019b).

#### Life Satisfaction

We used the five-item Satisfaction with Life Scale (Diener et al., 1985) to measure the global cognitive judgments of satisfaction with one’s life at W6. The agreement with the items had to be rated on a 7-point Likert-type scale of 1 (*strongly disagree*) to 7 (*strongly agree*). Cronbach’s alpha was 92. The structural validity of this scale was analyzed in a previous study using data from the same panel study (Udayar et al., 2020b).

#### Sociodemographic Variables

We used age, gender, household income, and education level as control variables in the hierarchical regression. Male participants were coded as 1 and females were coded as 2 at W6. Household income assessed the annual income of the household and was used as a continuous variable at W6 (1= *lowest annual income*, 8 = *highest annual income*). For the education level, participants had to indicate their education using a list of nine (nonordinal) options at the beginning of the panel study (W1). For the analytic purposes of the current study, we recategorized the responses into 1 (*higher education*), 2 (*secondary education*), and 3 (*primary education*).

### Statistical Analyses

For the purpose of cross-validating the LIVES-DHS, we randomly split the participants into two subsamples (respectively, *n*_1_ = 574 and *n*_2_ = 596). The two subsamples were similar with regard to gender, *t*(1168) = 0.08, *p* = .939, language *t*(1163.29) = 1.46, *p* = .146, and age, *t*(1086) = 1.01, *p* = .311. In the analyses presented in the current article, we coded language as 1 (*German*) and 2 (*French*), whereas we measured age as a continuous variable.

Using the first subsample (*n*_1_), we conducted the EFA. More specifically, we conducted a principal axis factor (PAF) analysis with an oblique rotation, to allow covarying multidimensional factors, in *R* to identify the factor structure of the LIVES-DHS. The extraction of factors was based on Eigenvalues (Eigenvalue > 1 being the criterion for extraction), scree plot, and parallel analysis. We selected the items based on the following criteria (Costello & Osborne, 2015): (a) items had to have a component loading of at least a .40 and not load on another factor at or above .30; (b) items within the factor had to be logically related to other items; and (c) each factor had to have at least three items.

Using the second subsample (*n*_2_), we conducted the CFA with a maximum likelihood to replicate and assess the structural validity of the solution emerging from the PAF. We tested and compared three different models: unidimensional model, five-factor model, and hierarchical model. We used the following fit indices to evaluate each model fit: the comparative fit index (CFI), the Tucker–Lewis index (TLI), the standardized root mean residual (SRMR), and the root mean square error of approximation (RMSEA). If the CFI was .90 or above, the TLI was above .95 and the SRMR and RMSEA were .08 or less, we considered the model to have a good fit (Cheung & Rensvold, 2002; Hu & Bentler, 1999). As the models are not nested, to compare them, we considered differences in the Bayesian Information Criterion (BIC) instead of calculating the chi-square difference.

We also tested measurement invariance across the two language groups based on the constrained versus unconstrained model differences in chi-square, CFI, RMSEA statistics, and the BIC. We considered noninvariance when model differences exceeded the recommended cutoff values (significant Δχ^2^, ΔCFI > .01, and ΔRMSEA > .015, lowest BIC, see Cheung & Rensvold, 2002; Putnick & Bornstein, 2016).

Finally, to examine concurrent validity, we calculated descriptive statistics, reliabilities, and bivariate correlations within the full sample (*N* = 1,170). We then conducted a series of hierarchical regressions to examine the relation between daily hassles and life satisfaction. Hence, in the first block of the model, we regressed life satisfaction on age, gender, household income, and education level. We added personality traits in the second block and daily hassles in the third block. We ran separate hierarchical regressions for the total score of the LIVES-DHS and for each subscale. We reported the nested model comparison using the *R*^2^ change *F*-test, the increments in *R*^2^, and whether these were statistically significant.

## Results

### Exploratory Factor Analysis

We conducted a PAF analysis with an oblique rotation. The Kaiser’s criterion suggested four factors, some of them being heterogeneous in terms of sources of daily hassles, whereas the scree plot and the parallel analysis suggested considering a five-factor solution, which corresponded to what was expected. For this reason, a five-factor solution with 20 items was preferred over the four-factor solution. The five-factor solution provided a well-defined factor structure, explaining 67% of the variance. Most items loaded on a single factor. Only three items showed cross-loadings over .30 (see Appendix 2, available in the online supplement). However, because the item “having to deal with health problems related to my work—for example, exhaustion or back problems” had a loading and a cross-loading of .40 and the item “not being recognized enough for what I do” was quite abstract, we discarded both of them first, keeping the item “having to deal with conflicts with my friends” which content was tangible and general enough. The new solution with 18 items provided a well-defined five-factor structure without cross-loadings (the cross-loading previously found with Item 9 disappeared) and explained 69% of the variance. We grouped the items by their hypothesized dimension, except Item 16. Given its wording (“Need unemployment benefits”), this item unsurprisingly loaded on the professional stress instead of the financial stress factor as originally assumed. Finally, correlations between the factors were inspected. They were moderate to high (between .43. and .63), suggesting the possibility of a higher order factor.

In sum, the results allowed us to retain 18 items in the LIVES-DHS, representing five factors that describe the type of hassles affecting everyday life. These hassles are similar to the theoretical model including, namely, physical (hassles related to physical and mental health problems; four items), professional (hassles related to job search and job insecurity; four items), relational (hassles related to conflicts with others; four items), environmental (hassles related to the various sources of insecurity of a country/place; three items), and financial aspects (hassles related to various everyday financial issues; three items). Hence, the obtained results showed conceptual support for the hypothesized daily hassles dimensions.

### Confirmatory Factor Analysis

We then tested the five-factor solution from the EFA with the CFA.^1^ The tested five-factor model exhibited acceptable fit indices: χ^2^(125) = 557.18, *p* < .001; CFI = .941; TLI = .928; RMSEA = .082, 90% confidence interval [.076, .090]; SRMR = .041; BIC = 16971.07. After comparing this five-factor model to a unidimensional model, it appeared that the five dimensions of daily hassles were meaningful sources of daily stress and provided superior fit to the unidimensional model. Factor loadings were also uniformly high in the five-factor model (Table 1). According to the BIC comparison of the five-factor model and the hierarchical model, the hierarchical model had the best fit (Table 2). Indeed, model fit indices were acceptable, and subscale factor loadings ranged from .75 to .87 for financial sources of daily hassles, from .51 to .89 for physical sources of daily hassles, from .59 to .84 for relational sources of daily hassles, from .80 to .89 for environmental sources of daily hassles, and from .81 to .92 for professional sources of daily hassles (Table 3). Although the five-factor model and hierarchical model showed quite similar fit indices, we kept the hierarchical model for the rest of the analyses as it was the closest one to the theoretical model.

**Table 1.** Factor Loadings of the LIVES Daily Hassles Scale After a Principal Axis Factor Analysis With an Oblimin Rotation.

Items	Sources of daily hassles				
	SF	SE	SPh	SR	SPr
1. Not having enough money to cover everyday expenses, such as paying bills, rent, or food.	**.62**	−.03	.05	.05	.10
2. Need the financial help of someone I know.	**.95**	.01	.00	.04	−.03
3. Need social assistance.	**.61**	.05	.15	.00	.17
4. Having to deal with a physical illness or mental health problem.	.05	−.04	**.84**	.06	.02
5. Being limited in my daily activities due to chronic illness or disability.	.06	.00	**.90**	−.05	.02
6. Facing the effects of aging.	−.15	.02	**.51**	.11	.06
7. Having to undergo important medical treatment.	−.01	.13	**.74**	.06	−.03
8. Having to deal with conflicts with other family members.	−.04	−.03	.17	**.57**	.07
9. Having to deal with conflicts with my friends.	.09	.07	.00	**.78**	.00
10. Being alone, without friends.	.09	.08	.06	**.62**	−.01
11. Having to deal with conflicts with colleagues at the workplace.	−.03	.04	.04	**.49**	.24
12. Become the victim of an assault/attack.	.03	**.79**	.01	.12	.03
13. Become the victim of theft or burglary.	.00	**.98**	.00	−.05	.00
14. Being affected in my health by environmental pollution.	−.05	**.49**	.18	.14	.05
15. Having to look for a job.	.09	.05	−.09	.02	**.83**
16. Need unemployment benefits.	.14	.08	.12	−.11	**.71**
17. Seeing my working conditions deteriorate—for example by a cut in wages or by the obligation to accept flexible hours.	−.02	−.02	.06	.12	**.71**
18. Losing my job.	−.06	.00	.02	.03	**.91**

*Note. N* = 574. Loadings ≤.40 in absolute value are in boldface and items’ labels are those of the LIVES-DHS. SF = financial source; SPh= physical source; SPr = professional source; SR = relational source; SE= environmental source.

**Table 2.** Fit Results of Structural Models of the LIVES Daily Hassles Scale.

18-Item models	χ ^2^	*df*	CFI	TLI	RMSEA	SRMR	BIC
1. Five-factor model	557.18^*^	125	0.941	0.928	0.082	0.041	16971.07
2. Unidimensional model	1667.53^*^	135	0.791	0.763	0.149	0.064	18019.11
3. Hierarchical model	584.62^*^	130	0.938	0.927	0.083	0.043	16967.36

*Note. df* = degrees of freedom; CFI = comparative fitness index; TLI = Tucker–Lewis index; RMSEA = root mean square error of approximation; SRMR = standardized root mean square residual; BIC = Bayesian Information Criterion.

* *p* < .001.

**Table 3.** Standardized Parameter Estimates From the Confirmatory Factor Analysis on the LIVES Daily Hassles Scale.

Items	1. Unidimensional model	2. Five-factor model					3. Hierarchical model				
		SF	SPh	SR	SE	SPr	SF	SPh	SR	SE	SPr
Item 1	.66	.75					.75				
Item 2	.75	.87					.88				
Item 3	.81	.87					.87				
Item 4	.76		.90					.89			
Item 5	.77		.93					.93			
Item 6	.46		.50					.51			
Item 7	.73		.81					.81			
Item 8	.54			.59					.59		
Item 9	.74			.83					.84		
Item 10	.71			.80					.80		
Item 11	.62			.68					.67		
Item 12	.79				.89					.89	
Item 13	.79				.88					.88	
Item 14	.74				.80					.80	
Item 15	.82					.92					.92
Item 16	.86					.91					.91
Item 17	.78					.81					.81
Item 18	.79					.85					.85
SF			.78	.77	.77	.80					
SPh				.74	.75	.68					
SR					.81	.80					
SE						.80					
DHs							.89	.83	.90	.89	.88

*Note. N* = 508. SF = financial source; SPh = physical source; SPr = professional source; SR = relational source; SE = environmental source; DHs = daily hassles.

Finally, we tested measurement invariance on the hierarchical model across the two language groups (i.e., German and French). Results showed support for the equivalence of factor loadings and intercepts across these two groups.

### Concurrent Validity of the LIVES-DHS

Table 4 shows the means, standard deviations, and correlations for all measures. Each of the LIVES-DHS factors correlated significantly with measures of related but different constructs. Specifically, the financial hassles was negatively correlated with income adequacy (*r* = −.56, *p* < .001), the physical hassles was negatively correlated with self-rated health (*r* = −.36, *p* < .001), the relational hassles was negatively correlated with social support (*r* = −.37, *p* < .001), and the professional hassles was positively correlated with job insecurity (*r* = .41, *p* < .001). All of these moderate correlations showed that the subscales of LIVES-DHS could differentiate the concept of daily hassles in a specific domain from associated concepts. Moreover, any given subscale of the LIVES-DHS always showed the highest correlation with the associated concept than with the other concepts. For example, financial hassles showed a higher correlation with income adequacy than with health, social support, and work stress, which also supports the concurrent validity. Concerning the environmental hassles, we lacked a specific construct to examine the concurrent validity. However, it was significantly correlated with all of the aforementioned constructs.

**Table 4.** Means, Standard Deviations, and Pearson Correlations (*N* =1,170).

Measures	*M*	*SD*	1	2	3	4	5	6	7	8	9	10	11	12	13	14	15	16	17	18	19	20	21	22
1. Daily hassles	1.71	0.68	1																					
2. SF	1.66	0.89	.82***	1																				
3. SPh	1.78	0.80	.87***	.63***	1																			
4. SR	1.72	0.69	.84***	.58***	.68***	1																		
5. SE	1.58	0.78	.82***	.55***	.65***	.68***	1																	
6. SPr	1.76	0.91	.85***	.68***	.63***	.60***	.63***	1																
7. Age	47.92	8.31	.06*	−.01	.12***	.02	.08**	.04	1															
8. Gender	1.51	0.50	.06	.03	.06*	.05	.09**	.01	−.03	1														
9. Education level	1.63	0.57	.18***	.21***	.13***	.10**	.16***	.18***	.08*	.00	1													
10. Household income	4.84	2.18	−.32***	−.41***	−.23***	−.20***	−.20***	−.29***	.02	−.08*	−.38***	1												
11. Income adequacy	3.21	0.90	−.45***	−.56***	−.35***	−.30***	−.25***	−.42***	.05	.01	−.25***	.60***	1											
12. Neuroticism	2.56	0.64	.36***	.28***	.33***	.34***	.26***	.32***	−.11***	.17***	.11**	−.25***	−.32***	1										
13. Extraversion	3.33	0.47	−.07*	−.06	−.09**	−.06	−.03	−.05	−.08**	.07*	−.19***	.17***	.08**	−.37***	1									
14. Openness	3.43	0.50	.03	.04	.02	.02	.05	.01	.08**	.11***	−.27***	.05	.03	−.05	.33***	1								
15. Agreeableness	3.62	0.44	−.00	.03	.01	−.03	.03	−.02	.12***	.19***	−.04	−.02	.01	−.17***	.19***	.21***	1							
16. Conscientiousness	3.84	0.44	−.09**	−.11**	−.09**	−.09**	−.01	−.07*	.02	.06*	.02	−.09**	.12***	−.37***	.28***	.06*	−25***	1						
17. Social support	4.11	0.86	−.34***	−.27***	−.30***	−.37***	−.16***	−.29***	.03	.08**	−.09**	.28***	.40***	−.39***	.21***	.09**	.15***	.19***	1					
18. Self-rated health	4.10	0.81	−.33***	−.28***	−.36***	−.26***	−.17***	−.30***	−.11***	.00	−.15***	.24***	.34***	−.35***	.23***	.07*	.11***	.17***	.34***	1				
19. Job insecurity	1.79	0.61	.24***	.19***	.12***	.14***	.12***	.41***	−.01	−.04	−.01	.15***	−.25***	.23***	−.09**	.01	−.14***	−.12***	−.19***	−.18***	1			
20. Life satisfaction	5.18	1.29	−.40***	−.40***	−.32***	−.37***	−.17***	−.39***	.03	.04	−.14***	.40***	.53***	−.48***	.29***	.06*	.10***	.26***	.57***	.49***	−.30***	1		
21. General mental health	0.97	0.50	.36***	.31***	.34***	.35***	.13***	.36***	−.03	.05	.06*	−.19***	.36***	.44***	−.21***	−.04	−.07*	−.20***	−.45***	−.50***	−.26***	−.58***	1	
22. Perceived stress in life	2.36	0.71	.42***	.35***	.35***	.39***	.22***	.41***	−.09**	.07*	.10**	−.28***	.39***	.56***	−.23***	−.05	−.10**	−.27***	−.48***	−.45***	−.26***	−.61***	.70***	1

*Note*. SF = financial source; SPh = physical source; Spr = professional source; SR = relational source; SE = environmental source.

* *p* < .05. **p < .01. ***p < .001.

The total score of daily hassles was positively correlated with neuroticism (*r* = .36, *p* < .001) and with perceived stress in life (*r* = .42, *p* < .001), and negatively correlated with general mental health (*r* = −.36, *p* < .001). It was also negatively correlated with life satisfaction (*r* = −.40, *p* < .001).

It is interesting to note that age was positively, albeit weakly, correlated with daily hassles, and especially with physical hassles (*r* = .12, *p* < .001) and environmental hassles (*r* = .08, *p* = .007). Similarly, gender was also positively, also weakly, correlated with the same sources of daily hassles: physical hassles (*r* = .06, *p* = .036) and environmental hassles (*r* = .09, *p* = .002).

### Relation Between Daily Hassles and Life Satisfaction

To examine the relation between daily hassles and life satisfaction while controlling for well-known covariates of it, we ran a series of hierarchical regressions in three steps (Table 5). As all five daily hassles subscales were highly correlated with each other (>.55), we conducted analyses for the overall score and each subscale separately to avoid multicollinearity. Results showed that the total score of daily hassles significantly predicted life satisfaction (β = −.23, *p* < .001). Although age, gender, and education level did not predict life satisfaction, household income predicted life satisfaction in the first block (accounting for 13.9% of the variance). Personality traits, especially neuroticism, extraversion, and conscientiousness, accounted for 18.1% of the variance in the second block, and overall score of daily hassles, which we added in the third block, accounted for an additional 4.3% of the variance. Results of hierarchical regressions with each subscales of the LIVES-DHS separately showed that financial hassles accounted for an additional 3.8% of the variance, physical hassles accounted for an additional 2% of the variance, professional hassles accounted for an additional 3.5% of the variance, relational hassles accounted for an additional 5.8% of the variance, and environmental hassles accounted for an additional 0.4% of the variance in the third block.

**Table 5.** Hierarchical Regression (*n* = 936): The Contribution of Daily Hassles (DH) to Life Satisfaction Beyond Age, Gender, Household Income, Education Level, and Personality Traits.

	Life satisfaction, β		
Independant variables	Block 1	Block 2	Block 3
Gender	.06	.11***	.10***
Age	.03	−.01	.01
Household income	.38***	.27***	.22***
Education level	.01	.03	.05
Neuroticism		−.38***	−.29***
Extraversion		.09**	.11***
Openness		.01	.02
Agreeableness		.00	.01
Conscientiousness		.06	.06*
DH-overall score			−.23***
*R* ^2^	.139	.320	.363
Δ*R*^2^		.181***	.043***
	Block 1	Block 2	Block 3
Gender	.06	.11***	.10***
Age	.03	−.01	.01
Household income	.38***	.27***	.20***
Education level	.01	.03	.05
Neuroticism		−.38***	−.33***
Extraversion		.09**	.11**
Openness		.01	.02
Agreeableness		.00	.01
Conscientiousness		.06	.05
DH-financial source			−.22***
*R* ^2^	.139	.320	.358
Δ*R*^2^		.181***	.038***
	Block 1	Block 2	Block 3
Gender	.06	.11***	.11***
Age	.03	−.01	.01
Household income	.38***	.27***	.25***
Education level	.01	.03	.04
Neuroticism		−.38***	−.33***
Extraversion		.09**	.10**
Openness		.01	.01
Agreeableness		.00	.01
Conscientiousness		.06	.06*
DH-physical source			−.16***
*R* ^2^	.139	.320	.340
Δ*R*^2^		.181***	.020***
	Block 1	Block 2	Block 3
Gender	.06	.11***	.10**
Age	.03	−.01	.01
Household income	.38***	.27***	.23***
Education level	.01	.03	.05
Neuroticism		−.38***	−.31***
	Life satisfaction, β		
	Block 1	Block 2	Block 3
Extraversion		.09**	.11***
Openness		.01	.02
Agreeableness		.00	.00
Conscientiousness		.06	.06*
DH-professional source			−.21***
*R* ^2^	.139	.320	.355
Δ*R*^2^		.181***	.035***
	Block 1	Block 2	Block 3
Gender	.06	.11***	.10***
Age	.03	−.01	.00
Household income	.38***	.27***	.24***
Education level	.01	.03	.04
Neuroticism		−.38***	−.29***
Extraversion		.09**	.11***
Openness		.01	.01
Agreeableness		.00	.01
Conscientiousness		.06	.06*
DH-relational source			−.26***
*R* ^2^	.139	.320	.378
Δ*R*^2^		.181***	.058***
	Block 1	Block 2	Block 3
Gender	.06	.11***	.11***
Age	.03	−.01	−.00
Household income	.38***	.27***	.26***
Education level	.01	.03	.04
Neuroticism		−.38***	−.36***
Extraversion		.09**	.10**
Openness		.01	.01
Agreeableness		.00	.00
Conscientiousness		.06	.06*
DH-environmental source			−.07*
*R* ^2^	.139	.320	.324
Δ*R*^2^		.181***	.004*

* *p* < .05. **p < .01. ***p < .001.

## Discussion

### LIVES Daily Hassles Scale: A Reliable Tool to Assess Daily Hassles

The current study aimed to validate a new scale assessing daily hassles that could be easily implemented in panel studies. The aim of the newly developed LIVES-DHS was to assess how central a hassle is for the person from a subjective point of view. We expected to reveal five sources of daily hassles as represented by a general higher order factor (Hypotheses 1a and 1b).

Overall, the LIVES-DHS showed good psychometric and structural properties. Based on the first random subsample, PAF analysis highlighted 18 items representing daily hassles that fell in five factors as expected. Using the second random subsample, CFAs indicated an acceptable fit for the 18-item hierarchical model, which confirmed our first hypothesis. The hierarchical structure with a general daily hassles factor and five sources of daily hassles fits the data well and meets measurement invariance requirements regarding the French- and German-speaking groups. In practical terms, its means that this scale could be used in three ways: by computing a total score, by using specific subscales separately, or all subscales together. The use of the scale would depend on the research question and the outcomes chosen. For example, if the study aims to examine the effect of daily hassles as a generalized source of stress on overall health and well-being beyond the effect of major life events, computing and using the total score could be enough. If the study aims to examine a specific source of daily hassles in a specific context (e.g., the role of professional sources of daily hassles in work-related well-being and productivity), then the subscale(s) of interest could be used separately. Finally, if the study aims to compare the effect of different sources of daily hassles and/or to examine which source of daily hassles explains the most the variance, then subscales could be considered altogether as long as multicollinearity has been checked and ruled out beforehand.

### Daily Hassles Are Related to, Yet Distinct From Other Similar Constructs

In a subsequent step, the current study aimed to examine the concurrent validity of the LIVES-DHS (Hypotheses 2a, 2b, and 2c) by computing bivariate correlations between four out of the five sources of daily hassles and theoretically relevant constructs, namely income adequacy, self-rated heath, social support, and job insecurity, between overall score of daily hassles and stress and health indicators (i.e., perceived stress in life and self-rated health), and between daily hassles and the personality dimension of neuroticism. As expected, the overall score of the LIVES-DHS and each of the LIVES-DHS sources of daily hassles correlated moderately with measures of related constructs. It means that our newly developed scale could differentiate the primary appraisal (importance) of daily negative events from outcomes related to those events (i.e., perceived stress and general mental health) and from the personality trait (i.e., neuroticism) associated with experiences of those events. These results show that LIVES-DHS is indeed a scale that assesses daily events through a subjective evaluation of them as being important in the person’s current life and not how much stressful they are. In this way, we also highlight the utility of such a tool that differentiates sources of daily stress from more global and general levels of stress and vulnerabilities. Income difficulties, health issues, lack of social hassles, and job insecurity all relate to daily hassles, even if we cannot say anything about the direction of the causality with this study (Bollmann et al., 2019).

### Daily Hassles Contribute to Lower Levels of Life Satisfaction

This study also aimed to examine the relation between daily hassles and life satisfaction by running a series of hierarchical regressions. Because daily hassles represent important sources of stress that people encounter in everyday life, we expected that they would predict a lower level of life satisfaction controlling for age, gender, household income, education level, and personality traits (Hypothesis 3). The results of the hierarchical regressions supported this third hypothesis: Daily hassles, both the total score and each source of daily hassles, significantly and negatively predicted life satisfaction and added significant incremental variance beyond that accounted for by age, gender, household income, and personality traits. In other words, when the participants were preoccupied by some daily events, they were less satisfied with their lives. These results support previous studies demonstrating the detrimental effect of daily hassles on well-being (e.g., Day et al., 2005; Hart, 1999; Lavee & Ben-Ari, 2008; Wright et al., 2010) and are in line with the theoretical reasoning about the harmful nature of psychological stressors (Lazarus & Folkman, 1984). Most important, our findings showed that this negative effect on well-being was still present once the effects of the well-known covariates of life satisfaction were controlled for.

These results, together with those obtained from factor analyses, converge with the stress spillover theory (Pearlin & Bierman, 2013). Specifically, they demonstrate that people who were preoccupied by some daily events in one specific domain seemed to also worry about daily events in other domains, which could explain why daily hassles negatively predicted the satisfaction with life in general.

Previous studies have shown that interpersonal hassles are more stress-inducing than other types of hassles (e.g., Bolger et al., 1989; McIntyre et al., 2008). Similarly, in our study, relational hassles had a stronger effect on life satisfaction compared with other sources of daily hassles. Forming and maintaining good relationships with others is one core psychological need to satisfy to have a fulfilled life (e.g., Baumeister & Leary, 1995; Haslam et al., 2016). Empirical studies have highlighted the major role of social relationships in individuals’ satisfaction with life (Erdogan et al., 2012), and especially those of supportive relationships. Hence, it is not surprising that concerns about conflictual relationships might play a key role in life satisfaction.

### Practical Applications

We believe that this newly validated tool for assessing daily hassles could benefit both research and practice. Its relatively short length makes the LIVES-DHS easier to implement in panel studies than long checklists and open ended questions. Moreover, the multidimensionality of this new scale makes it also a versatile tool for research. Notably, most of the existing tools are designed to assess either general levels of stress in life, measuring how many various situations in one’s life have been appraised as stressful over a given period (e.g., the Perceived Stress Scale; Cohen et al., 1983), or strain tied to one particular domain, measuring for instance individuals’ experience of stress at work (e.g., the General Work Stress Scale; De Bruin & Taylor, 2005). As a result, there is a lack of comprehensive tools that would allow researchers to measure different daily stressors in various life domains at the same time. With increasing attention to spillover processes between two or more domains (e.g., Bernardi et al., 2017; Spini et al., 2017; van Emmerik et al., 2016), having such a multidimensional measure is of great importance. Hence, we expect that the LIVES-DHS may serve this purpose by focusing on five interrelated yet different groups of stressors, thereby helping further researchers investigate stress spillover effects and similar phenomena.

From a practical point of view, this scale could also be useful applied contexts. For example, in career counseling, when working with counselees who feel stressed out or express career-related indecision, it could be used as a tool to point out and understand the concerns or difficulties faced at the time by the counselees. It could also identify the sources of daily hassles that wear down individuals and prevent them from making a career decision. For example, in the case of adults in retraining, this scale could provide interesting information about the challenges that contribute to their career stagnation. Thus, identifying the stressors that people are most concerned about could help both the counselor and counselee understand where the problems are and start to thinking about solutions. In sum, it may lead to deeper reflections about daily hassles in one’s life, thereby promoting a more reality-based career or, more generally, life construction process.

### Limitations and Future Directions

Since the LIVES-DHS is a new tool to assess daily hassles, its psychometric properties must be cross-validated across different (cultural) contexts. Thus, it would be important to test the hierarchical five-factor structure stability and the measurement invariance of the 18-item solution in other samples or countries. This could be particularly relevant considering this scale focuses on the most salient groups of stressors in a given context; the list might well not be generalizable to other national or cultural contexts. Hence, it is possible that other important groups of stressors or factors exist, and they may be added to the existing ones in the future. It is also important to note that this scale could be subject to retrospective recall and memory biases, although these effects should be less pronounced than those we could expect in checklists as individuals had to report how much they worried about a specific hassle “today.”

As the data used in the current study were cross-sectional, there is always a possibility that life satisfaction contributes to changes in concerns about daily hassles and not vice versa. For instance, people with a lower level of life satisfaction might be more easily preoccupied by daily events because they may be more sensitive to negative experiences. Therefore, further research is needed to examine the direction of these effects. We used a score of life satisfaction in this study, which is an indicator of subjective well-being. Future studies may also focus on the relation between daily hassles and well-being using an objective approach and more varied indicators. Similarly, futures studies should also examine the association of the LIVES-DHS with other related constructs, such as major life events, and with other observational measures of minor hassles (e.g., with The Daily Inventory of Stressful Events; Almeida et al., 2002).

## Conclusion

In conclusion, the LIVES-DHS appears to be a psychometrically valid instrument to measure daily hassles. Its 18-item version should be suitable for and easily implemented in large surveys, which resolves the issues with long checklists and open-ended questions. Due to its relatively short length, the current scale could also be an interesting supplement for practitioners to assess and follow-up on counselees’ daily hassles in different domains.

## Supplemental Material

sj-pdf-1-asm-10.1177_10731911211047894 – Supplemental material for The LIVES Daily Hassles Scale and Its Relation to Life Satisfaction Click here for additional data file. Supplemental material, sj-pdf-1-asm-10.1177_10731911211047894 for The LIVES Daily Hassles Scale and Its Relation to Life Satisfaction by Shagini Udayar, Ieva Urbanaviciute, Davide Morselli, Grégoire Bollmann, Jérome Rossier and Dario Spini in Assessment

sj-pdf-2-asm-10.1177_10731911211047894 – Supplemental material for The LIVES Daily Hassles Scale and Its Relation to Life Satisfaction Click here for additional data file. Supplemental material, sj-pdf-2-asm-10.1177_10731911211047894 for The LIVES Daily Hassles Scale and Its Relation to Life Satisfaction by Shagini Udayar, Ieva Urbanaviciute, Davide Morselli, Grégoire Bollmann, Jérome Rossier and Dario Spini in Assessment

## Notes

1. Beauducel and Herzberg (2006) point out that weighted least square mean and variance adjusted is more appropriate than maximum likelihood when dealing with fewer than five response categories. Hence, we did the analyses and as they are virtually identical, we decided to report maximum likelihood results because of the BIC.
