# Supplementary material for: The LIVES Daily Hassles Scale and Its Relation to Life Satisfaction
Source: Assessment. 2021 Oct 18;30(2):348–63. doi: 10.1177/10731911211047894 (PMC9902985; doi:10.1177/10731911211047894)
Supplement: sj-pdf-1-asm-10.1177_10731911211047894 – Supplemental material for The LIVES Daily Hassles Scale and Its Relation to Life Satisfaction [file sj-pdf-1-asm-10.1177_10731911211047894.pdf]

Appendix 1

The final 18-item LIVES Daily Hassles Scale in French and German.

n°item	Sources	item wordings
1	Financial	Ne pas avoir suffisamment d’argent pour couvrir les dépenses courantes, par exemple pour payer des factures, le loyer ou la nourriture.<br>Nicht über genügend Geld zu verfügen, um die laufenden Kosten zu begleichen, z.B. Rechnungen, die Miete und Nahrungsmittel zu bezahlen.<br>Not having enough money to cover everyday expenses, such as paying bills, rent or food.
2	Financial	Avoir besoin de l’aide financière d’une personne de mon entourage.<br>Auf finanzielle Hilfe von jemandem aus meiner näheren Umgebung angewiesen zu sein.<br>Need the financial help of someone I know.
3	Financial	Avoir besoin de l’aide sociale.<br>Auf Sozialhilfe angewiesen zu sein.<br>Need social assistance.
4	Physical	Devoir faire face à une maladie ou un problème de santé mentale.<br>Sich mit einer Krankheit oder einer geistigen Erkrankung zurechtfinden müssen.<br>Having to deal with a physical illness or mental health problem.
5	Physical	Être diminué dans mes activités quotidiennes à cause d’une maladie chronique ou d’une invalidité.<br>In meinen täglichen Aktivitäten aufgrund einer chronischen Erkrankung oder Invalidität eingeschränkt zu sein.<br>Being limited in my daily activities due to chronic illness or disability.
6	Physical	Devoir faire face aux effets du vieillissement.<br>Sich mit den Folgen des Älterwerdens zurechtfinden müssen.<br>Facing the effects of aging.
7	Physical	Devoir suivre un traitement médical important.<br>Sich einer bedeutenden medizinischen Behandlung unterziehen müssen.<br>Having to undergo important medical treatment.
8	Relational	Devoir faire face à des conflits avec d’autres membres de la famille.<br>Sich mit Konflikten mit Familienmitgliedern auseinandersetzen müssen.<br>Having to deal with conflicts with other family members.
9	Relational	Devoir vivre des tensions avec mes ami-e-s.<br>Spannungen mit meinen Freunden ausgesetzt zu sein.<br>Having to deal with conflicts with my friends.
10	Relational	Me retrouver seul-e, sans ami-e-s.<br>Alleine zu sein, ohne Freunde.<br>Being alone, without friends.
11	Relational	Devoir vivre des tensions avec des personnes dans le cadre de mon travail.<br>Spannung mit Personen auf meiner Arbeit zu erleben.<br>Having to deal with conflicts with colleagues at the workplace.

12	Environmental	Devenir victime d'une agression.<br>Opfer eines Angriffs zu werden.<br>Become the victim of an assault/attack.
13	Environmental	Devenir victime d'un vol ou d'un cambriolage.<br>Opfer eines Diebstahls oder Einbruchs zu sein.<br>Become the victim of theft or burglary.
14	Environmental	Être atteint-e dans ma santé par la pollution ambiante.<br>Gesundheitliche Probleme im Zusammenhang mit der Umweltverschmutzung zu haben.<br>Being affected in my health by environmental pollution.
15	Professional	Devoir chercher un emploi.<br>Eine Arbeit suchen zu müssen.<br>Having to look for a job.
16	Professional	Avoir besoin des indemnités de chômage.<br>Arbeitslosenhilfe zu brauchen.<br>Need unemployment benefits.
Only for employed people :		
17	Professional	Voir mes conditions de travail se détériorer, par exemple par une baisse de salaire ou par l'obligation d'accepter des horaires flexibles.<br>Zu sehen, wie sich meine Arbeitsbidungungen verschlechtern, zum Beispiel eine Lohnsenkung oder die Verpflichtung flexible Arbeitszeiten zu akzpetieren.<br>Seeing my working conditions deteriorate—for example by a cut in wages or by the obligation to accept flexible hours.
18	Professional	Perdre mon emploi.<br>Meine Arbeit zu verlieren.<br>Losing my job.

Note. *Some items not retained in this study could be relevant in another context or situation. Readers interested in these items can contact the corresponding author to obtain their French and German versions.*
