# Supplementary material for: The LIVES Daily Hassles Scale and Its Relation to Life Satisfaction
Source: Assessment. 2021 Oct 18;30(2):348–63. doi: 10.1177/10731911211047894 (PMC9902985; doi:10.1177/10731911211047894)
Supplement: sj-pdf-2-asm-10.1177_10731911211047894 – Supplemental material for The LIVES Daily Hassles Scale and Its Relation to Life Satisfaction [file sj-pdf-2-asm-10.1177_10731911211047894.pdf]

*Appendix 2*

*Factor Loading of the LIVES Daily Hassles Scale After a Principal Axis Factor Analysis With an Oblimin Rotation (20 items).*

| | Sources of Daily Hassles | | | | |
|---|---|---|---|---|---|
| | Fin. | Env. | Phys. | Relat. | Prof. |
| Item 1 | **.66** | -.02 | .05 | .02 | .08 |
| Item 2 | **.93** | -.01 | .00 | .03 | -.01 |
| Item 3 | **.64** | .05 | .15 | -.01 | .16 |
| Item 4 | .06 | -.05 | **.84** | .05 | .03 |
| Item 5 | .07 | -.01 | **.89** | -.06 | .02 |
| Item 6 | -.14 | .01 | **.52** | .14 | .07 |
| Item 7 | 0.01 | .16 | **.75** | .01 | -.04 |
| Item 8 | .04 | .14 | .24 | **.39** | -.02 |
| Item 9 | .20 | **.32** | .13 | **.46** | -.12 |
| Item 10 | .18 | .24 | .12 | **.47** | -.09 |
| Item 11 | .03 | .10 | .04 | **.55** | .21 |
| Item 12 | .08 | -.07 | .02 | **.53** | **.32** |
| Item 13 | .04 | **.83** | .00 | .08 | .04 |
| Item 14 | -.02 | **.92** | -.01 | -.05 | .05 |
| Item 15 | -.04 | **.55** | .20 | .07 | .05 |
| Item 16 | .17 | .14 | -.06 | -.01 | **.71** |
| Item 17 | .19 | .12 | .13 | -.14 | **.64** |
| Item 18 | .00 | -.03 | .07 | .20 | **.72** |
| Item 19 | .02 | .08 | .05 | .02 | **.79** |
| Item 20 | -.13 | .07 | **.40** | .16 | **.40** |

*Note*. $N = 574$. Loadings $\leq$0.40 and cross-loadings $\leq$0.30 in absolute value are in boldface and items' labels are those of the LIVES-DHS.
